# Complete mitochondrial DNA sequence of the European flat oyster *Ostrea edulis *confirms Ostreidae classification

**DOI:** 10.1186/1756-0500-4-400

**Published:** 2011-10-12

**Authors:** Gwenaelle Danic-Tchaleu, Serge Heurtebise, Benjamin Morga, Sylvie Lapègue

**Affiliations:** 1 IFREMER, Laboratoire de Génétique et Pathologie, F-17390 La Tremblade, France

## Abstract

**Background:**

Because of its typical architecture, inheritance and small size, mitochondrial (mt) DNA is widely used for phylogenetic studies. Gene order is generally conserved in most taxa although some groups show considerable variation. This is particularly true in the phylum Mollusca, especially in the Bivalvia. During the last few years, there have been significant increases in the number of complete mitochondrial sequences available. For bivalves, 35 complete mitochondrial genomes are now available in GenBank, a number that has more than doubled in the last three years, representing 6 families and 23 genera. In the current study, we determined the complete mtDNA sequence of *O. edulis*, the European flat oyster. We present an analysis of features of its gene content and genome organization in comparison with other *Ostrea*, *Saccostrea *and *Crassostrea *species.

**Results:**

The *Ostrea edulis *mt genome is 16 320 bp in length and codes for 37 genes (12 protein-coding genes, 2 rRNAs and 23 tRNAs) on the same strand. As in other Ostreidae, *O. edulis *mt genome contains a split of the *rrnL *gene and a duplication of *trnM*. The tRNA gene set of *O. edulis*, *Ostrea denselamellosa *and *Crassostrea virginica *are identical in having 23 tRNA genes, in contrast to Asian oysters, which have 25 tRNA genes (except for *C. ariakensis *with 24). *O. edulis *and *O. denselamellosa *share the same gene order, but differ from other Ostreidae and are closer to *Crassostrea *than to *Saccostrea*. Phylogenetic analyses reinforce the taxonomic classification of the 3 families Ostreidae, Mytilidae and Pectinidae. Within the Ostreidae family the results also reveal a closer relationship between *Ostrea *and *Saccostrea *than between *Ostrea *and *Crassostrea*.

**Conclusions:**

*Ostrea edulis *mitogenomic analyses show a high level of conservation within the genus *Ostrea*, whereas they show a high level of variation within the Ostreidae family. These features provide useful information for further evolutionary analysis of oyster mitogenomes.

## Background

Because of its typical architecture, inheritance and small size, animal mitochondrial (mt) DNA is widely used for phylogenetic studies. Combined with these characteristics, its typically maternal inheritance contributes to a fast rate of evolution. Nucleotide changes combined with gene order and rearrangement data can provide valuable information on major evolutionary changes at different taxonomic levels. Typically, animal mtDNA is a compact molecule (14 to 17 kb), though some mtDNA can be vastly larger (e.g., *Plactopecten magellanicus *[1]), and usually encodes 13 proteins, 22 transfer RNAs (tRNAs) and 2 ribosomal RNAs (rRNAs) [2]. There are often few intergenic nucleotides except for a single large non-coding region generally thought to contain elements that control the initiation of replication and transcription [3]. Size variation in mtDNA is usually due to the different length of the non-coding regions. Gene order is generally conserved in most taxa, although some groups show considerable variation. This is particularly so in the Mollusca phylum, especially in Bivalvia and Scaphopoda [4]. In addition to the fact that phylogenetic relationships among major molluscan groups are not well understood, the species classification of some of the most common mollusks remains difficult.

A case in point is oysters, for which a plastic growth pattern is a major feature, resulting in a wide range of overlapping ecophenotypic variants [5,6]. Oysters are bivalve molluscs that are widely distributed in the world's oceans. As benthic, sessile filter-feeders, they play an important role in estuarine ecosystems. Moreover, some species are of economic importance, like the Pacific cupped oyster, which is grown in 27 countries and is the most highly produced mollusc species in the world. Oysters have been introduced all over the world for culture and many species are sympatric. Numerous species (30-40 according to the classifications) of oysters of the genus *Ostrea *have been described. Their geographical range is particularly wide in warm and temperate waters of all oceans, although they have a predominantly tropical distribution [6,7]. In Europe, along the Atlantic and Mediterranean coasts, the European flat oyster, *Ostrea edulis*, is an important economic marine resource: in 2009 almost 3000 tons were produced in the world, mainly (91%) in Europe (Spain, France, Ireland ...) [8].

During the last few years, there have been significant increases in the number of complete mitochondrial sequences available for all species. The number has more than doubled for molluscs in the last three years [9], so that 98 complete mollusk mitochondrial genomes are now available in GenBank, mainly from gastropods (43), bivalves (35) and cephalopods (14). Among bivalves, the sequenced genomes represent 6 families and 23 genera. In the Ostreidae, the genus *Crassostrea *has been thoroughly studied, with 7 representatives (6 Asian oysters and 1 American oyster) [10]. In contrast, there is only one representative of the genus *Saccostrea *(*Saccostrea mordax*), and one of the genus *Ostrea *(*Ostrea denselamellosa*). Recent studies have provided a more comprehensive picture of the cupped oyster genome, showing an unusually high conservation of mitochondrial gene order in Asian *Crassostrea *species [11]. Even though molecular tools, such as mitochondrial or microsatellite markers, already exist for the European flat oyster and allow population genetics [12] or quantitative genetics [13] studies, the complete characterization of its mtDNA will allow a better study to be made of phylogenetic relationships among members of the genus, especially between the closely-related species *O. edulis *and *O. angasi *[14], to improve classification of the Ostreidae family within the Bivalvia.

## Results and discussion

### Genome composition

**Figure 1:**
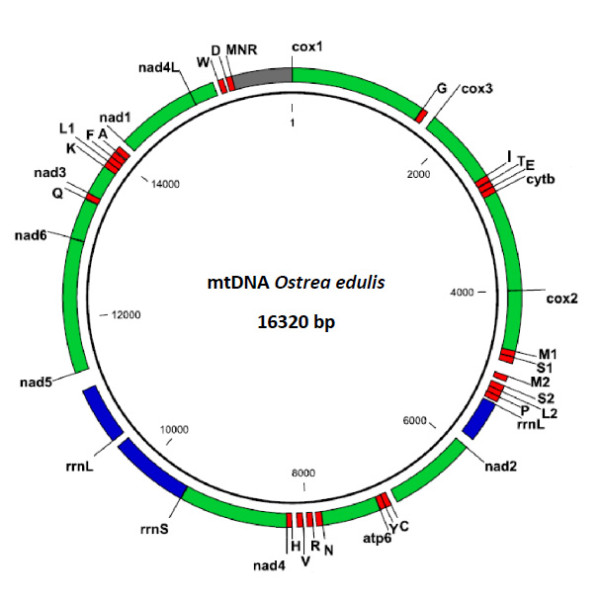
**Mitochondrial genome map of *Ostrea edulis***. Genes for proteins (green) and rRNAs (blue) are abbreviated with standard abbreviations. The one letter amino acid code is used for tRNA (red) designation. The major non-coding region (MNR) is shown in grey.

The complete mitochondrial genome of *Ostrea edulis *[GenBank: JF274008] is 16 320 nt in length and encodes 37 genes, including 12 protein-coding genes (PCGs), 2 rRNAs and 23 tRNAs on the same strand (Figure 1 and Table 1). This size is very close to that of *O. denselamellosa *(16 277 bp), shorter than that of other Ostreidae (16 532 bp for *S. mordax *to 22 446 bp for *C. iredalei*), and is within the size range of the Pteriomorphia mt genomes published to date: from 16 211 nt for *Argopecten irradians *[15] to 32 115 nt for *Placopecten magellanicus *[1].

**Table 1:** Features of *Ostrea edulis *mitochondrial genome.

Feature	Sequence location	Size	Start codon	Stop codon	Intergenic region*
cox1	1-1566	1566	ATG	TAA	6
trnG	1573-1639	67			95
cox3	1735-2622	888	ATA	TAG	-1
trnI	2622-2687	66			10
trnT	2698-2761	64			7
trnE	2769-2836	68			-6
cytb	2831-3997	1167	CTA	TAA	1
cox2	3999-4691	693	ATG	TAA	3
trnM1	4695-4759	65			7
trnS1	4767-4836	70			151
trnM2	4988-5051	64			51
trnS2	5103-5172	70			1
trnL1	5173-5239	67			1
trnP	5241-5304	64			17
rrnL 5'end	5322-5896	575			25
nad2	5922-6929	1008	ATT	TAA	73
trnC	7003-7066	64			3
trnY	7070-7134	65			6
atp6	7141-7809	669	ATA	TAG	3
trnN	7813-7883	71			14
trnR	7898-7964	67			4
trnV	7969-8035	67			27
trnH	8063-8126	64			-15
nad4	8112-9476	1365	ATA	TAG	0
rrnS	9477-10411	935			81
rrnL 3'end	10493-11200	708			172
nad5	11373-12920	1548	ATG	TAA	7
nad6	12928-13395	468	ATA	TAA	9
trnQ	13405-13470	66			1
nad3	13472-13825	354	ATG	TAG	-1
trnK	13825-13891	67			3
trnL2	13895-13960	66			1
trnF	13962-14028	67			13
trnA	14042-14106	65			79
nad1	14186-15118	933	ATG	TAA	1
nad4L	15120-15401	282	ATG	TAA	60
trnW	15462-15524	63			33
trnD	15558-15625	68			0
MNR	15626-16320	695			0

*Negative numbers indicate overlapping nucleotides between adjacent genes

In the mt genome of *O. edulis*, a total of 965 bp of non-coding nucleotides is spread over 21 intergenic regions (each over 5 bp) including a major non-coding region (MNR) of 695 bp. A large non-coding region suggests a putative control region based on its AT content of 74.4% [16]. In contrast to typical animal mitochondrial genomes, the *O. edulis *genome may lack the protein-coding gene *atp8*, although some recent studies have found a good candidate for *atp8 *gene in Mytilidae and possibly in some Ostreidae [17]. Furthermore, *O. edulis *genome also has duplications of three tRNAs: *trnM*, *trnS *and *trnL*. The *rrnL *gene is split into 2 fragments, a phenomenon previously observed in the Ostreidae [11]. The *rrnS *is not duplicated (also in *O. denselamellosa*, *S. mordax *and *C. virginica*), in contrast to Asian *Crassostrea*.

The molecule has an overall A+T composition value of 64.9% and the size of the coding region is 15 379 nt in length, accounting for 94.2% of the whole genome. The AT content is slightly higher than those of Pectinidae (55.3 to 59.6% [18]) or Mytilidae (61.5 to 61.8%). The AT composition of *O. edulis *is, therefore, within the AT content range of the Ostreidae: the lowest known AT content is 60.7% in *O. denselamellosa*, while the highest is 65.3% in *C. hongkongensis *[19]. In *S. mordax*, the AT content is 64.4% which is very similar to *O. edulis*.

As observed in *O. denselamellosa *(16 277 bp), *S. mordax *(16,532 bp) and *C. virginica *(17 244 bp), the lack of duplicated *rrnS *in *O. edulis*, added to the lack of 2 tRNAs (not duplicated *trn-K *and *trn-Q*) compared to Asian *Crassostrea *may account for the difference in length compared with other *Crassostrea *(*C. gigas *18225 bp, *C. hongkongensis *18 622 bp).

The genome composition of *O. edulis *is, thus, identical to *O. denselamellosa *(except for AT composition) and close to *S. mordax *in terms of complete genome size, AT content and the non-duplicated *rrnS*. More mitochondrial genome sequences from *Ostrea *and *Saccostrea *will be needed to assess relationships between the *Ostrea*, *Crassostrea *and *Saccostrea genera*.

### Gene arrangement

**Figure 2:**
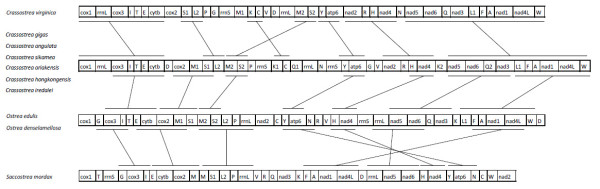
**The gene arrangement map of Ostreidae mitochondrial genomes**. The bars show identical gene blocks. All genes are transcribed from left-to-right.

Animal mt gene order is relatively stable within major groups and generally variable among groups [2]. Bivalve species show variability in terms of mt genome size, gene arrangement and tRNA number [20]. As observed in four Pectinidae (*A. irradians*, *M. yessoensis*, *C. farreri *and *P. magellanicus*), gene arrangement can be very different despite species being members of the same family [15]. In contrast, in *Mytilus *congeners *M. edulis*, *M. trossulus *and *M. galloprovincialis*, PCGs are arranged in an identical order, but tRNA, rRNA and control regions are also almost the same [21]. The mt gene order of *O. edulis *is identical to that of *O. denselamellosa *(Figure 2). The arrangement of PCGs in *O. edulis *is *cox1*, *cox3*, *cytb*, *cox2*, *nad2*, *atp6*, *nad4*, *nad5*, *nad6*, *nad3*, nad1, *nad4L *and is nearly identical to that of C*rassostrea*, except for the inversion of two PCGs *nad2 *and *atp6*. Among the six Asian *Crassostrea *and their Atlantic sister species *C. virginica*, only protein-coding gene order is identical [10]. Besides, gene order of *Crassostrea *differs significantly from *S. mordax*. Ostreidae share three PCG blocks *cox1-cox3-cytb-cox2*, *nad5-nad6 *and *nad3-nad1-nad4L*. Moreover, the *nad2-atp6-nad4 *block of *O. edulis *is inverted in *S. mordax *(Figure 2), but the remaining genes are extensively rearranged. The major non-coding region (*MNR) *is located after *trnD *(Figure 1), while this region is found between *trnG *and *trnV *near *atp6 *and *nad2 *in *Crassostrea*. If tRNAs and rRNAs are considered, there are six blocks conserved within the Ostreidae: *cox3-trnI, cox2-trnM, trnM-trnS, trnY-atp6, trnH-nad4 *and *trnF-trnA-nad1-nad4L*. Between *O. edulis *and Asian *Crassostrea*, seven blocks are shared: *cox3-trnI-trnT-trnE-cytb*, *cox2-trnM-trnS, trnM-trnS, trnY-atp6, trnH-nad4, nad5-nad6-trnQ-nad3 *and *trnL-trnF-trnA-nad1-nad4L-trnW*; while between *O. edulis *and *S. mordax*, seven blocks are also shared but these are different: *trnG-cox3-trnI, trnE-cytb-cox2-trnM, trnM-trnS-trnL-trnP-rrnL(5'end)*, *trnF-trnA-nad1-nad4L, rrnL(3'end)-nad5-nad6, trnH-nad4 *and *trnY-atp6*. It should be noted that *rrnL *is in one piece in *Saccostrea *but not within *Crassostrea*.

In terms of gene arrangement, it is thus clear that *O. edulis *is more similar to *Crassostrea *than to *S. mordax *when comparing PCGs. As shown in Figure 2, the complete genome arrangement of *O. edulis *is similar to that of Asian *Crassostrea *while it appears completely reorganized from *trnY *to the end of mt genome when compared with that of *S. mordax*.

### Protein-coding genes

All PCGs are encoded on and transcribed from the same strand. Twelve open reading frames (ORFs) were detected for the thirteen typical PCGs (*cox1-cox3, cytb, nad1-nad6, nad4L, atp6 *and *atp8*). Although we carefully looked for candidate regions for *atp8 *gene, we could not identify any, as in all Pteriomorphia complete genomes already published. However, a recent publication [17] suggests that a putative ORF represents a good candidate to start an *atp8 *gene in most bivalve mt genomes. Within the invertebrate mt code there are three standard initiation codons (M-AUG, M-AUA, and I-AUU), but mt genomes often use a variety of non conventional start codons [22]. In this study, most of PCGs use conventional initiation codons: ATA is used for *cox3, nad4, nad6 *and *atp6*; ATG is used for *cox1, cox2, nad1, nad3*, *nad4L *and *nad5*; ATT is used for *nad2*, but *cytb *uses the alternative start codon CTA (as in *C. gigas *and *C. angulata *[10]). Eight protein-coding genes were terminated by a stop codon (TAA and TAG).

### Transfer and ribosomal RNA genes

In total, 23 tRNA coding genes were identified in the size range of 63 to 71 nucleotides, based on typical secondary structure (Additional file 1). An additional *trnM *was detected as found in *C. gigas*, *C. hongkongensis *[9], *C. virginica *[16] and *Mytilus *[23]. Two serine and two leucine tRNA genes were also differentiated in *O. edulis *by their anticodons (UCA Ser1, AGA Ser2, and CUA Leu1, UUA Leu2) as found in *O. denselamellosa *[24], *Crassostrea *and some other species (*M. edulis*, *M. galloprovincialis *and *Argopecten irradians*). The anticodon usage of *O. edulis *was congruent with the corresponding tRNA genes of other molluscan mtDNAs.

Identification of both the small and the large ribosomal RNA genes in *O. edulis *was accomplished by BLAST comparison with other published ribosomal RNA genes, especially *O. denselamellosa *[GenBank:HM015199], *S. mordax *[GenBank:FJ841968] and *C. gigas *[GenBank:EU672831]. Although putative gene boundaries for the two rRNA genes have been found, these cannot be precisely determined until transcript mapping is carried out. Besides *rrnS *of *O. edulis *is 935 bp in length and flanked by *nad4 *and *rrnL 3'end*.

The *rrnL *gene is split into two segments: one segment, of the 5' end (matches with *rrnL 5'end *from *O. denselamellosa *and *Saccostrea*), is 575 bp long and positioned between *trnP *and *nad2*; and the other segment, of the 3' end, is 708 bp and located between *rrnS *and *nad5*. The length of the *rrnS *is similar to that of most bivalves, but smaller than that of *O. denselamellosa *(1017 bp) and that of *Crassostrea *(946 to 1207 bp) [10]. The size of *rrnL *(1283 bp in all) is similar to that of *O. denselamellosa *(1299 bp), but smaller than that of other bivalves. This bias may be due to the method (BLAST) used to compare the rRNA sequences because this method only checks the identity between a few sequences and because it's easier to compare sequences from same species as they show higher identity.

### Non-coding regions

As in most bivalves, *O. edulis *mtDNA contains a large number of unassigned nucleotides. There are as many as 21 non-coding regions (> 5 bp) up to 965 nucleotides found throughout the *O. edulis *mitochondrial genome. Eight of these non-coding regions are more than 50 bp in length. Among these regions, the major non-coding region (MNR) has been identified and located, that remains the most promising region in which to find regulatory and/or gender-specific sequences [25]. The *O. edulis *mtDNA MNR is positioned between *trnD *and *cox1 *and is 695 bp in length, similar to that of *O. denselamellosa *(689 bp), making it the longest MNR within the Ostreidae apart from *C. virginica *(832 bp) and *C. ariakensis *(716 bp). It has an A+T content of 74.4% which is higher than the remainder of the mt genome (64.4%), as it includes several (A)n and (T)n homopolymer tracts, features which are typically used for identification of the mitochondrial control region and thought to contain the replication origin [2].

### Phylogenetic analysis

In recent years there have been many phylogenetic studies on the taxonomy and evolution of the Ostreidae based on molecular data, especially mitochondrial DNA [26-30]. However, most of these previous studies have been based on partial sequences and incomplete molecular information. Recently, Ren et al. [11] have compared 7 complete mt genomes from Asian oysters.

**Figure 3:**
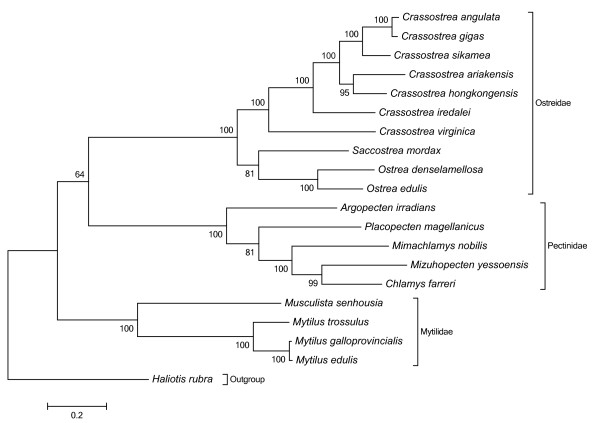
**Phylogenetic tree based on twelve concatenated PCGs from 19 mitochondrial genomes**.

**Figure 4:**
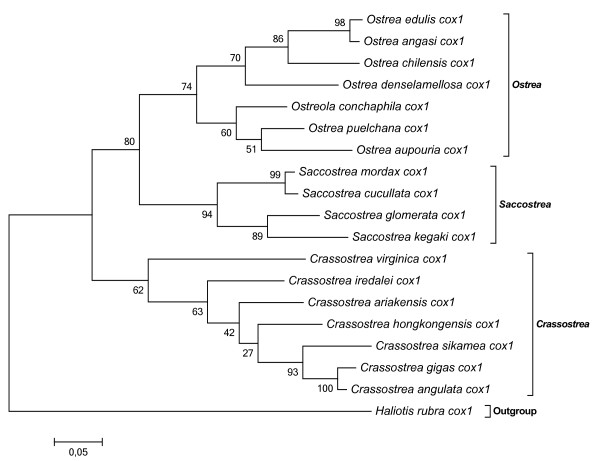
**Phylogenetic tree based on cox1 from all published *Ostrea*, *Ostreola*, *Saccostrea *and *Crassostrea***.

In the present study's aa-based tree built with twelve concatened PCGs from 19 mitochondrial genomes in Pteromorphia (Figure 3), we can observe that, at the Ostreidae level, *O. edulis *is first clustered with *O. denselamellosa *as congeneric species. Then this group of species falls into a highly supported clade with *S. mordax. Ostrea *and *Saccostrea *are then clustered with the *Crassostrea *species group. In this latest clade, the single American oyster *C. virginica *falls at the base of a nested clade that contains the Asian oysters. Very similar results were obtained with a nucleotide phylogenetic tree with low differences of bootstrap values. In Figure 4, more Ostreidae species are included as more numerous cox1 sequences are available in Genbank. The same phylogenetic relationship between *Ostrea*, *Saccostrea*, and *Crassostrea *is observed, especially the first grouping of *Ostrea *and *Saccostrea*, but not between *Ostrea *and *Crassostrea*, with however far less robust nodes. This same result was observed when considering the evolution of the tRNA anticodons in marine bivalve mitochondrial genomes, where the relationship presented are also based on concatenated nucleotide sequences of 12 protein-coding genes by Bayesian inference analysis [24]. However a recent study [31], based on cox1 and 16S sequences, showed a closer relationship between *Ostrea *and *Crassostrea*, than with *Ostrea *and *Saccostrea*. However, for the cox1 analysis, only one *Ostrea *sequence was included, and for the 16S analysis, much more *Ostrea *sequences were included but the bootstrap value was between 50 and 80%. Those comparisons seem to indicate that phylogenetic analyses are more powerful when including several sequences as the 12 concatened PCGs.

Finally, the phylogenetic tree presented in Figure 3, which includes mt genomes from all published Pteriomorphia, reinforces the taxonomic classification of the 3 families Ostreidae, Mytilidae and Pectinidae [32,11].

## Conclusion

In conclusion, the complete mitochondrial genome of *O. edulis *is 16 320 bp in length. A common phenomenon is that mitogenomes of most bivalves contain two *trnM *genes and most metazoan mitochondria have a set of 22 tRNA, including two *trnL *and two *trnS*. However the tRNA gene sets of *O. edulis*, *O. denselamellosa *and *C. virginica *are identical in having 23 tRNA genes. Another important characteristic is that the *rrnS *gene is not duplicated in *O. edulis*, a feature shared with *O. denselamellosa*, *S. mordax *and *C. virginica *and which contrasts with Asian *Crassostrea*.

The phylogenetic analyses confirm the relationships between each family (Ostreidae, Mytilidae and Pectinidae), but also within each genus (*Ostrea*, *Saccostrea *and *Crassostrea*). Within the Ostreidae, phylogenetic analyses show that *Ostrea *are closer to *Saccostrea *than *Crassostrea*, although gene arrangement may show a closer relationship between *Ostrea *and *Crassostrea*, indicating that several types of information are needed to infer relationships between genome species as evolution is acting at different levels of the genomes. As many questions remain unanswered on the phylogeny of Ostreidae, especially between *Ostrea *and *Saccostrea*, it would be desirable to increase the resolution by adding samples of more taxa in order to extend molecular information among the major lineages of the Ostreidae and within the Pteriomorphia as a whole.

## Methods

### PCR amplification and DNA sequencing

Adductor muscle from three *O. edulis *collected in Quiberon Bay (Bretagne, France) was used in this study. Total genomic DNA was extracted using a Wizard^®^DNA Clean-up System (Promega). The mitochondrial genome was amplified in 4 overlapping fragments using species-specific primers (Additional file 2). PCR was performed in 25 μl reaction volumes in a thermocycler (Applied Biosystems). Each reaction contained 13.3 μl dH2O, 5.0 μl buffer 5× (Promega), 2.0 μl MgCl2 (25 mM), 2.5 μl dNTP (2 mM), 0.5 μl of each primer (20 μM), and 0.2 μl GoTaq^®^DNA polymerase (5U/μl, Promega). PCR cycling conditions were 94 °C for 2 min; then 30 cycles of 94 °C for 30 sec, 57 °C for 30 sec and 72 °C for 2 min; and finally a step of 72 °C for 10 min. PCR products were verified by electrophoresis (1% agarose gel) and purified using Montage^®^PCR Centrifugal Filter Devices (Millipore). Purified products were then used directly as templates in cycle sequencing reactions with dyelabeled terminators (Big Dye 3.1, Applied Biosystems). Specific primers were designed and used for primer walking sequencing, which was performed for both strands of each sample on an ABI 3130XL/Genetic Analyser (ABI).

### Sequence analysis and gene annotation

During the processing of large fragments and those from primer walking sequencing, regular and manual examinations were used to ensure there was reliable overlapping and correct genome assembly.

Protein-coding and ribosomal RNA genes were firstly identified using BLAST [33] searches at GenBank, and then by alignment with previously published mt genomes from species of *Crassostrea*, *Saccostrea *and other closely-related molluscs. Amino-acid sequences of protein-coding genes were inferred with ORF Finder [34] using invertebrate mitochondrial genetic code. Transfer RNAs were identified using DOGMA [35]http://dogma.ccbb.utexas.edu/, and tRNAscan-SE [36]http://selab.janelia.org/tRNAscan-SE/ using mito/chloroplast genetic code and default search mode, or setting the cove cutoff score to 1 when necessary. Assembly of the genome and gene map of the mitochondrial genome of *Ostrea edulis *was performed using CLC Main Workbench (CLC bio).

### Phylogenetic analysis

To date, 20 Pteriomorphia mt genomes are available in GenBank [37] and we used 19 of these (excluding *Argopecten irradians irradians *that is very close to *Argopecten irradians*: 99% similarity) in our phylogenetic analysis, together with *O. edulis *mt genome obtained in this study (Table 2). The blacklip abalone *Haliotis rubra *(Gastropoda) was used as the outgroup. The nucleotide and amino-acid sequences from all 12 PCGs (protein-coding genes) were concatenated for each genome and aligned using MUSCLE [38]. The size of the concatenated alignment nucleotides varied from 10 411 bp for *M. yessoensis *to 11 240 bp for *P. magellanicus*). To the alignments, we applied a Maximum Likelihood (ML) phylogenetic reconstruction approach using 100 bootstraps with MEGA5 [39]. A second phylogenetic analysis was performed using 5 additional *cox1 *genes from *Ostrea *(*Ostrea angasi *[GenBank:AF112287.1], *Ostrea aupouria *[GenBank:AF112288.1], *Ostrea chilensis *[GenBank:AF112289.1], *Ostrea puelchana *[GenBank:DQ226521.1], and *Ostreola conchaphila *[GenBank:DQ464125.1]) and 3 from *Saccostrea *(*Saccostrea cucullata *[GenBank:AY038076.1], *Saccostrea glomerata *[GenBank:EU007483.1] and *Saccostrea kegaki *[GenBank:AB076910.1]).

**Table 2:** List of complete mitogenomes used in this study.

Tax on	Classification	**GenBank Accession Number**	Size
**Mollusca**			
**Bivalvia**			
**Pteriomorphia**			
*Mytilus edulis*	Mytiloida; Mytiloidea; Mytilidae	AY484747	16,740 nt
*Mytilus galloprovincialis*	Mytiloida; Mytiloidea; Mytilidae	AY497292	16,744 nt
*Mytilus trossulus*	Mytiloida; Mytiloidea; Mytilidae	AY823625	18,652 nt
*Musculista senhousia*	Mytiloida; Mytiloidea; Mytilidae	GU001954	20,612 nt
*Crassostrea angulata*	Ostreoida; Ostreoidea; Ostreidae	EU672832	18,225 nt
*Crassostrea ariakensis*	Ostreoida; Ostreoidea; Ostreidae	EU672835	18,414 nt
*Crassostrea gigas*	Ostreoida; Ostreoidea; Ostreidae	EU672831	18,225 nt
*Crassostrea hongkongensis*	Ostreoida; Ostreoidea; Ostreidae	EU672834	18,622 nt
*Crassostrea iredalei*	Ostreoida; Ostreoidea; Ostreidae	FJ841967	22,446 nt
*Crassostrea sikamea*	Ostreoida; Ostreoidea; Ostreidae	EU672833	18,243 nt
*Crassostrea virginica*	Ostreoida; Ostreoidea; Ostreidae	Y905542	17,244 nt
*Saccostrea mordax*	Ostreoida; Ostreoidea; Ostreidae	FJ841968	16,532 nt
*Ostrea denselamellosa*	Ostreoida; Ostreoidea; Ostreidae	HM015199	16,277 nt
*Ostrea edulis*	Ostreoida; Ostreoidea; Ostreidae	JF274008	16,320 nt
*Argopecten irradians*	Pectinoida; Pectinoidae; Pectinidae	EU023915	16,221 nt
*Chlamys farreri*	Pectinoida; Pectinoidae; Pectinidae	EU715252	21,695 nt
*Mizuhopecten yessoensis*	Pectinoida; Pectinoidae; Pectinidae	AB271769	20,414 nt
*Placopecten magellanicus*	Pectinoida; Pectinoidae; Pectinidae	DQ088274	32,115 nt
*Mimachlamys nobilis*	Pectinoida; Pectinoidea; Pectinidae	FJ415225	17,963 nt
**Gastropoda**			
**Vetigastropoda**			
*Haliotis rubra*	Haliotoidea; Haliotidae	NC_005940	16,907 nt

## Competing interests

The authors declare that they have no competing interests.

## Authors' contributions

GDT, SH and BM obtained the sequences. GDT and SL performed analyses and wrote the first draft of the publication. All the co-authors finalised the manuscript.

## Supplementary Material

Additional file 1 **The potential secondary structures of 22 tRNAs of *Ostrea edulis***. The duplication of methionine is named M1 and M2 respectively. Codons recognized are shown for the pairs of leucine (L1 and L2) and serine (S1 and S2). Click here for file

Additional file 2 Primers used for amplification of 4 large fragments in mitochondrial genome of *Ostrea edulis*. Click here for file
