# Supplementary figures and images for: Complete mitochondrial DNA sequence of the European flat oyster Ostrea edulis confirms Ostreidae classification
Source: BMC Res Notes. 2011 Oct 12;4:400. doi: 10.1186/1756-0500-4-400 (PMC3214155; doi:10.1186/1756-0500-4-400)

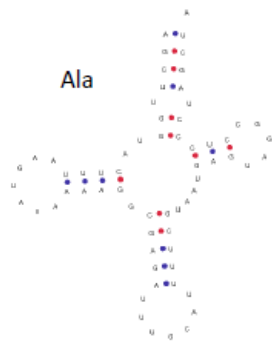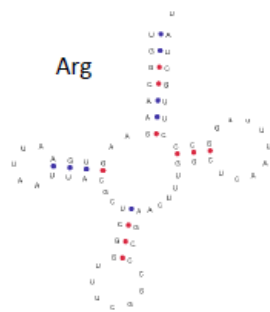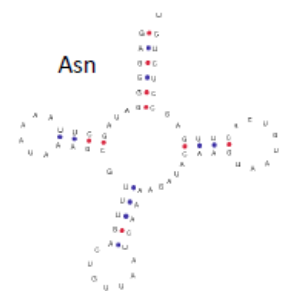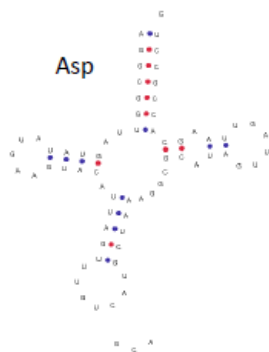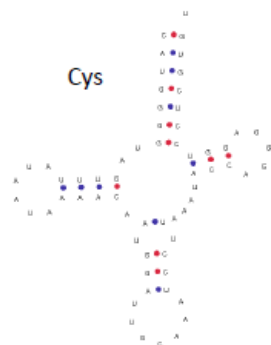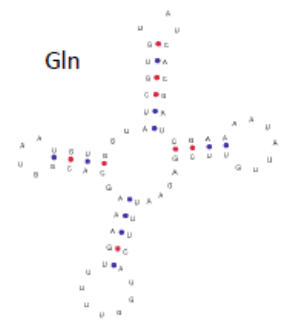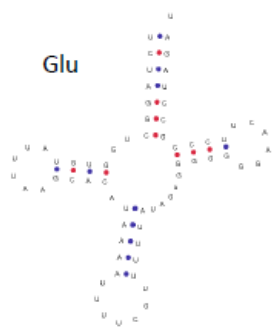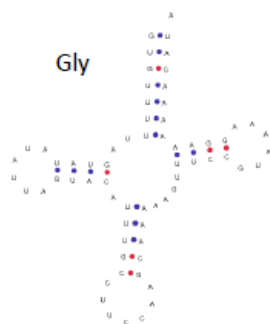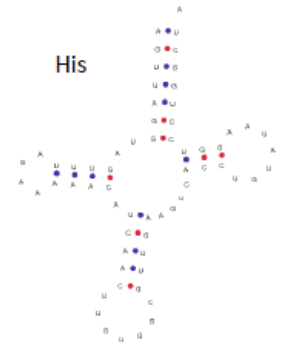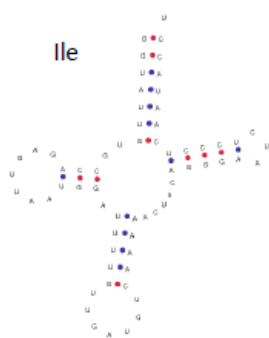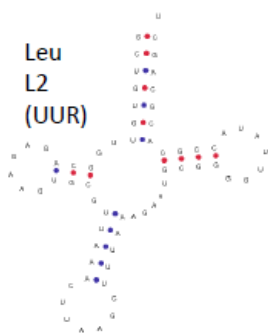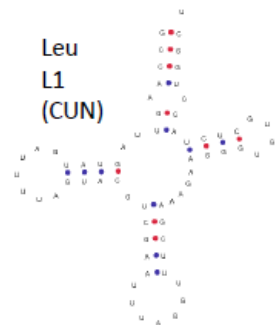

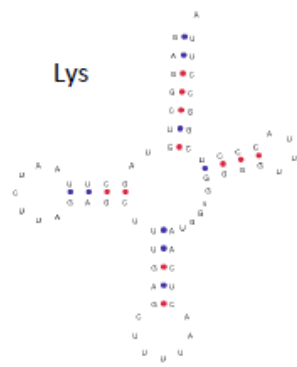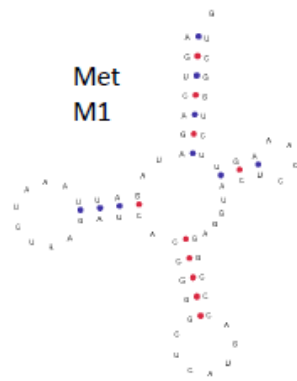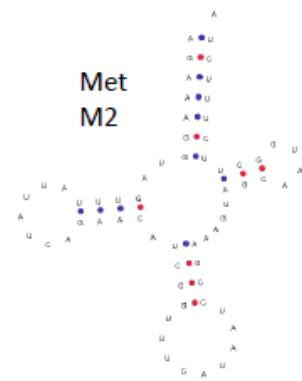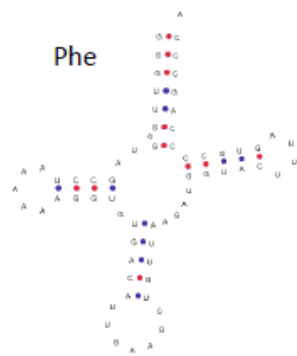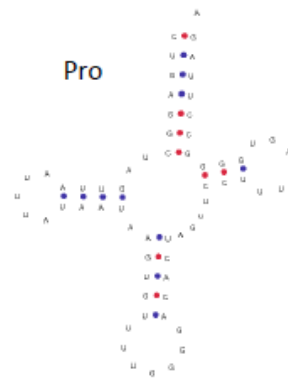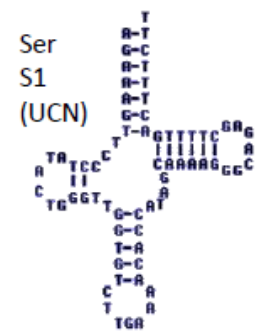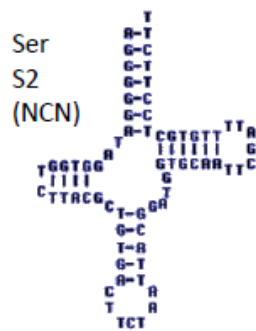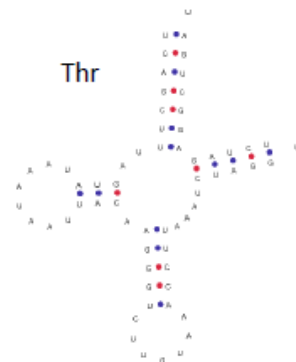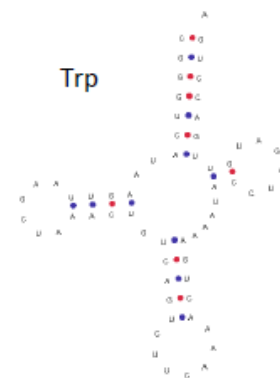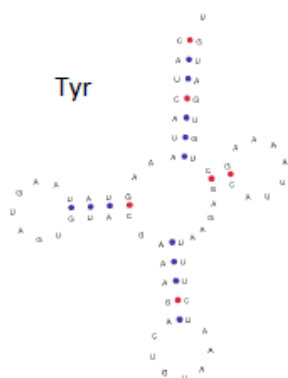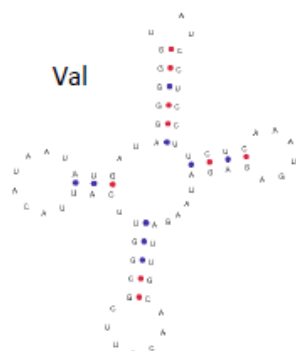

Supplement: Additional file 1 — The potential secondary structures of 22 tRNAs of Ostrea edulis. The duplication of methionine is named M1 and M2 respectively. Codons recognized are shown for the pairs of leucine (L1 and L2) and serine (S1 and S2). [file 1756-0500-4-400-S1.PDF]
