# Supplementary material for: Complete mitochondrial DNA sequence of the European flat oyster Ostrea edulis confirms Ostreidae classification
Source: BMC Res Notes. 2011 Oct 12;4:400. doi: 10.1186/1756-0500-4-400 (PMC3214155; doi:10.1186/1756-0500-4-400)
Supplement: Additional file 2 — Primers used for amplification of 4 large fragments in mitochondrial genome of Ostrea edulis. [file 1756-0500-4-400-S2.PDF]

Additional file 2 : Primers used for amplification of 4 large fragments in mitochondrial genome of *Ostrea edulis*.

Primer name	Sequence (5'-3')	Positions	Product size (bp)
CO1F	TGCTGTGGTCACAATACATGC	219-239	5383
16S5R	TCAACCAGCTATCCTAAGTCTCG	5579-5601	
CytbF	GACTTAGTAGGGTTTGCTTTTGGA	3530-3553	4193
ATP6R	CACCAGCTCCCAGATTATCAC	7702-7722	
ATP6E	GGAGCTGGTGGTAATATTGGTTC	7713-7735	5074
NAD5R	CCTCTGCTATATTTTCCCCTTC	12765-12786	
NAD5E	TTAGACTCGGTATCTCGATCTGC	12699-12721	4344
CO1R	CCACCACCTACAGGGTCAAA	703-722	
